# EDIN-B Promotes the Translocation of *Staphylococcus aureus* to the Bloodstream in the Course of Pneumonia

**DOI:** 10.3390/toxins7104131

**Published:** 2015-10-15

**Authors:** Johan Courjon, Patrick Munro, Yvonne Benito, Orane Visvikis, Coralie Bouchiat, Laurent Boyer, Anne Doye, Hubert Lepidi, Eric Ghigo, Jean-Philippe Lavigne, François Vandenesch, Emmanuel Lemichez

**Affiliations:** 1 INSERM U1065, Equipe Labellisée Ligue Contre le Cancer, Centre Méditerranéen de Médecine Moléculaire (C3M), Université de Nice Sophia-Antipolis, Nice 06204, France; E-Mails: johan_courjon@hotmail.fr (J.C.); munro@unice.fr (P.M.); Orane.Visvikis@unice.fr (O.V.); boyerl@unice.fr (L.B.); doye@unice.fr (A.D.); 2 CIRI, International Center for Infectiology Research, Inserm U1111, Université Lyon 1, Ecole Normale Supérieure de Lyon, CNRS UMR5308, 7 Rue Guillaume Paradin, Lyon 69372, France; E-Mails: yvonne.benito@chu-lyon.fr (Y.B.); coralie.bouchiat@chu-lyon.fr (C.B.); francois.vandenesch@univ-lyon1.fr (F.V.); 3 CNRS UMR 7278, IRD198, INSERM U1095, Aix-Marseille Université, 27 Bd Jean Moulin, Marseille 13385, France; E-Mails: Hubert.Lepidi@ap-hm.fr (H.L.); eric.ghigo@univ-amu.fr (E.G.); 4 Service de Microbiologie, CHU Carémeau, Place du Professeur Robert Debré, Nîmes 30029, France; E-Mail: jphlavigne@hotmail.com

**Keywords:** bacteremia, virulence factor, toxins, methicillin-resistant *Staphylococcus aureus*

## Abstract

It is crucial to define risk factors that contribute to host invasion by *Staphylococcus* *aureus*. Here, we demonstrate that the chromosomally encoded EDIN-B isoform from *S. aureus* contributes to the onset of bacteremia during the course of pneumonia. Deletion of *edin*B in a European lineage community-acquired methicillin resistant *S. aureus* (CA-MRSA) strain (ST80-MRSA-IV) dramatically decreased the frequency and magnitude of bacteremia in mice suffering from pneumonia. This deletion had no effect on the bacterial burden in both blood circulation and lung tissues. Re-expression of wild-type EDIN-B, unlike the catalytically inactive mutant EDIN-R185E, restored the invasive characteristics of ST80-MRSA-IV.

## 1. Introduction

*Staphylococcus aureus* is emerging as a major etiological agent of bacteremia and invasive infections. Bacteremia occurring during the course of pneumonia provoked both an increase of morbidity and mortality [1,2]. *S. aureus* produces an impressive arsenal of diverse virulence factors [3]; however with the exception of host cell adhesion molecules, little is known regarding the factors that promote bacterial translocation across epithelial and endothelial barriers [4,5].

Several pathogenic strains of *S. aureus*, particularly those of the European community-acquired methicillin-resistant *S. aureus* (MRSA) lineage (ST80-MRSA-IV) and others [6], express the epidermal cell differentiation inhibitor (EDIN) or EDIN-like exotoxins [7,8,9]. EDIN exotoxins belong to the family of C3 exoenzymes from *Clostridium botulinum* [7,8,9,10]. This group of factors catalyzes the ADP-ribosylation and inactivation of the small GTPase RhoA [7,11,12] and, to a lesser extent other Rho proteins [13]. Unlike other EDIN-like factors, EDIN-B is encoded on the chromosome [9,14].

The actin cytoskeleton and upstream regulators, notably the Rho GTPases, are common targets of major virulence factors expressed by highly pathogenic bacteria [10,15,16]. These small GTPases of the Rho protein family are essential proteins owing to their capacity to control the actin cytoskeleton, and therefore, the architecture of cells and tissues [15,17]. Cell biology approaches have revealed that ADP-ribosyltransferases targeting RhoA contribute to notch intercellular junctions in the epithelia, promote infection of gut tissues, as well as the formation of transcellular tunnels in endothelial cells, thereby compromising host barriers [5,15,18,19]. The *in vivo* relevance of EDIN factors during infection remains largely unappreciated.

Here, we constructed an *edin*B deletion mutant in a well-characterized strain of the ST80-MRSA-IV lineage and complemented strains to study the effect of EDIN activity in a mouse model of pneumonia.

## 2. Results

### 2.1. edinB Promotes Translocation of S. aureus into the Bloodstream During Pneumonia

**Figure 1:**
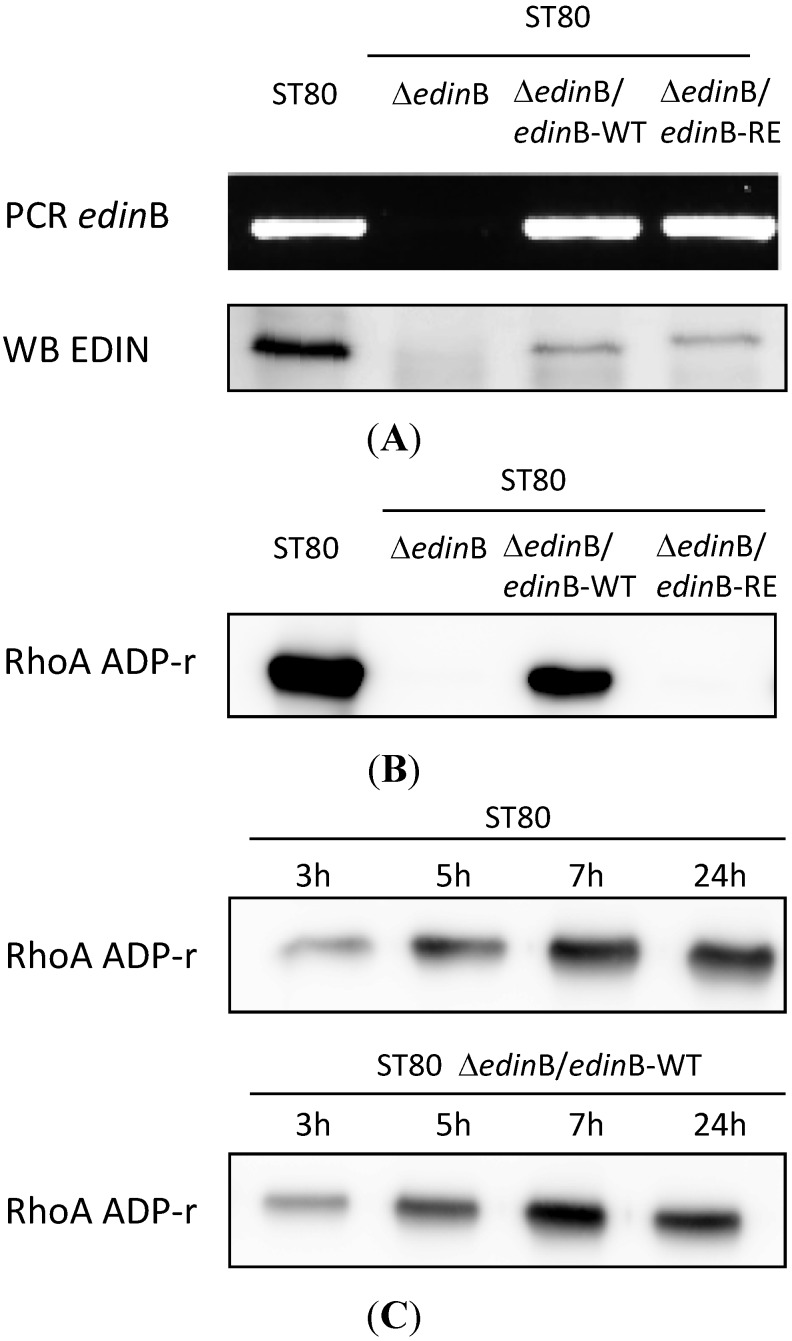
Characterization of the ST80 and ST80 derived strains. (**A**) Genetic and functional analysis of the different strains. PCR and Western blot (WB) showing the presence or absence of *edin*B and EDIN-B in the WT strain ST80 *SCCmecIV* PVL + MRSA (ST80), the isogenic strain deleted from *edin*B (Δ*edin*B) and the Δ*edin*B strain complemented with expression plasmids encoding EDIN-B WT (Δ*edin*B/*edin*B-WT) or the catalytically inactive mutant R185E (Δ*edin*B/*edin*B-RE); (**B**,**C**) Detection of the ADP-ribosyltransferase activity targeting RhoA in the supernatant of ST80 stains grown 24 h (**B**) or 3, 5, 7, and 24 h (**C**). The biotinylated form of ADP-ribosylated RhoA was revealed with peroxidase-coupled streptavidin.

We deleted the *edin*B gene from WT ST80-MRSA-IV strain to generate the Δ*edin*B isogenic strain (Δ*edin*B) (Figure 1A). Moreover, we generated expression plasmids for EDINB wild-type (EDIN-WT) and the catalytically inactive mutant R185E (EDIN-RE). We complemented the Δ*edin*B strain with each plasmid to generate Δ*edin*B/*edin*B-WT and Δ*edin*B/*edin*B-RE strains (Figure 1A). The presence or absence of the *edin*B-encoding gene and toxin expression was verified in the different strains by PCR and immunoblotting (Figure 1A). Next, we tested the ADP-ribosylation activity of EDIN associated with the supernatant of the different strains. This confirmed the production of an ADP-ribosyltransferase activity targeting RhoA by WT ST80-MRSA-IV strain (ST80) and the Δ*edin*B/*edin*B-WT strains opposite to Δ*edin*B and Δ*edin*B/*edin*B-RE strains (Figure 1B). In parallel, we measured an increase of the RhoA ADP-ribosylation activity in the supernatant of EDIN-B producing strains as a function of time (Figure 1C). Altogether these data validated at the genetic and functional levels the deletion and complementation of EDIN-B in the different strains.

**Figure 2:**
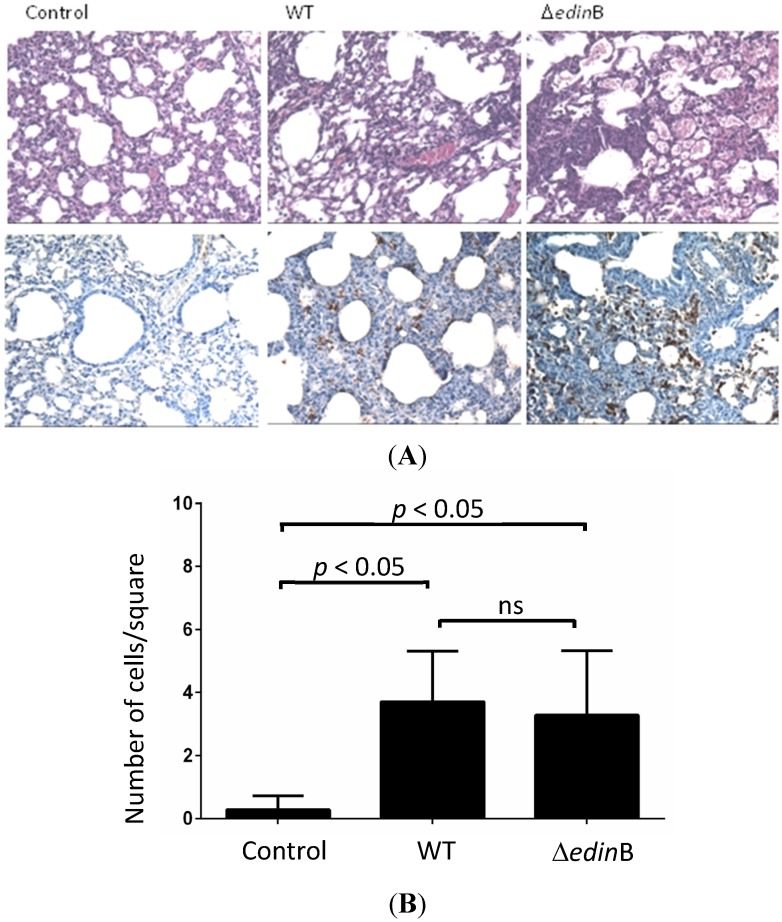
Histopathological examination. (**A**) Histopathological examination of the lung tissues of control, WT- and Δ*edin*B-infected mice showing thickened alveolar walls heavily infiltrated with immune cells (higher panels), as well as an immunohistochemical demonstration of bacteria within inflammatory infiltrates (lower panels). Magnification 40×; (**B**) Quantification of inflammatory infiltrates. Cells were scored manually using the ImageJ plugin cell counter. Cells were counted on image squares of 88 mm × 88 mm (*n* = 25 squares from 3 different mice; *p* < 0.05 or ns, not significant).

We then triggered pneumonia in mice with both the WT and Δ*edin*B strains. Pathological examination of the lung tissues showed inflammatory infiltrates largely confined within interalveolar walls mainly by a mononuclear inflammatory infiltrate composed of macrophages and lymphocytes, with a few neutrophils. Some bronchioalveolar air spaces were filled with rare alveolar macrophages and neutrophils (Figure 2A). Immunohistochemical analysis confirmed the presence of bacteria in lung tissues of both WT and Δ*edin*B infected mice (Figure 2A). Quantification of inflammatory infiltrates showed no significant difference between WT and Δ*edin*B strains as opposed to the control condition (Figure 2B).

**Figure 3:**
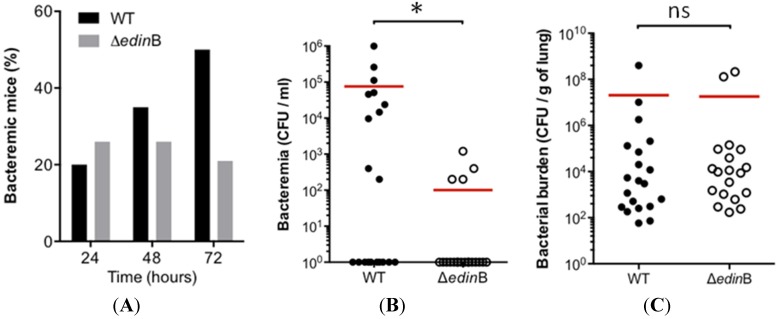
Role of *edin*B in pneumonia and bacteremia triggered by ST80-MRSA-IV. (**A**) Histogram showing the percentage of bacteremic mice after intranasal inoculation of 8 × 10^9^ CFU of the WT and Δ*edin*B strains (20 and 19 animals, respectively); (**B**) Groups of mice were infected by intranasal inoculation of 8 × 10^9^ CFU of the WT and Δ*edin*B strains for 72 h (*n* = 20 and 19, respectively). Dots represent the CFU values in the blood for each animal; red bars show the mean values; * *p* = 0.0159; (**C**) Groups of mice (*n* = 20 and 19, respectively) were infected by intranasal inoculation of 8 × 10^9^ CFU of the WT and Δ*edin*B strains for 72 h. Dots represent the CFU values in the lung tissues for each animal; red bars show the mean values (ns, not significant).

In parallel we monitored the occurrence and magnitude of bacteremia during the first 72 h of pneumonia (Figure 3A). In the WT strain-infected mice (*n* = 20), we measured a progressive increase in the occurrence of bacteremia, reaching 50% of the population at 72 h post-inoculation. In the Δ*edin*B-infected mice (*n* = 19), the percentage of concurrent bacteremia remained stable over the study period, reaching no more than 26% of the infected animals (Figure 3A). We went on to assess whether this higher occurrence of bacteremic animals in WT infected mice might be associated with higher bacterial burden in the blood. Indeed, we measured that bacteremic animals infected with the WT strain had a higher bacterial load in the blood of 7.6 × 10^4^ CFU/mL compared to 1 × 10^2^ CFU/mL for those infected with the Δ*edin*B strain (*p* = 0.0159) (Figure 3B). In contrast, we recorded no significant differences of bacterial burden in the lung tissues between both groups of animals (Figure 3C). Altogether, these results demonstrate that both the frequency and magnitude of bacteremia increase as a function of the presence of *edin*B.

Taken together these data revealed the critical role of EDIN-B in bacterial translocation into the bloodstream.

### 2.2. EDIN-B Activity Promotes the Translocation of S. aureus into the Bloodstream during Pneumonia

**Figure 4:**
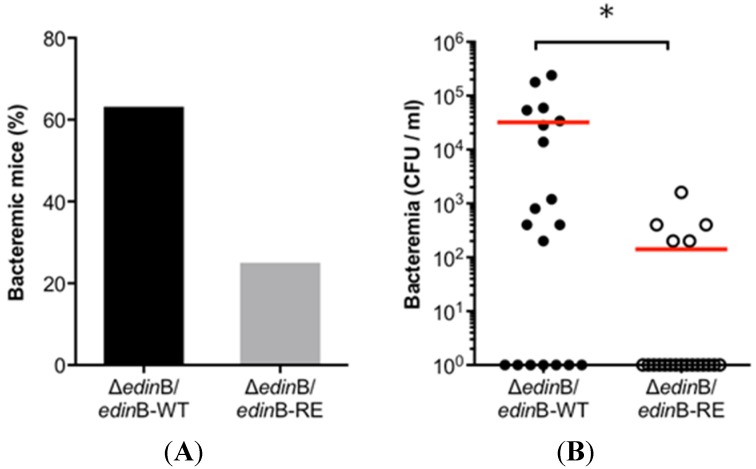
EDIN-B activity promotes bacterial translocation into the bloodstream during pneumonia. Groups of mice were infected for 48 h by intranasal inoculation of 9 × 10^9^ CFU of the Δ*edin*B/*edin*B-WT or Δ*edin*B/*edin*B-RE strains (*n* = 19 and *n* = 20, respectively). (**A**) Histogram showing the percentage of bacteremic mice; (**B**) Dots represent the CFU values in the blood for each animal; red bars show the mean values (* *p* = 0.035).

Next, we investigated the link between this newly described virulence property of EDIN-B and its enzymatic activity. To this aim, we generated the Δ*edin*B/*edin*B-WT and Δ*edin*B/*edin*B-RE strains. We triggered pneumonia in groups of mice with each strain. Of note, we measured a stability of both plasmids during the first 48 h of infection (not shown). We observed that the percentage of bacteremic animals reached 63% in the group of animals infected with Δ*edin*B/*edin*B-WT (*n* = 19), compared to 25% with Δ*edin*B/*edin*B-RE infected mice (*n* = 20) (Figure 4A). In parallel, we measured a dramatic increase of bacterial burden in the blood of animals infected with Δ*edin*B/*edin*B-WT (3.2 × 10^4^ CFU/mL) compared to Δ*edin*B/*edin*B-RE (1.4 × 10^2^ CFU/mL) (*p* = 0.0035) (Figure 4B).

These results show that the catalytically active EDIN-B potentiates the capacity of *S. aureus* to translocate into the bloodstream.

### 2.3. EDIN-B Activity and the Bacterial Persistence in the Blood

**Figure 5:**
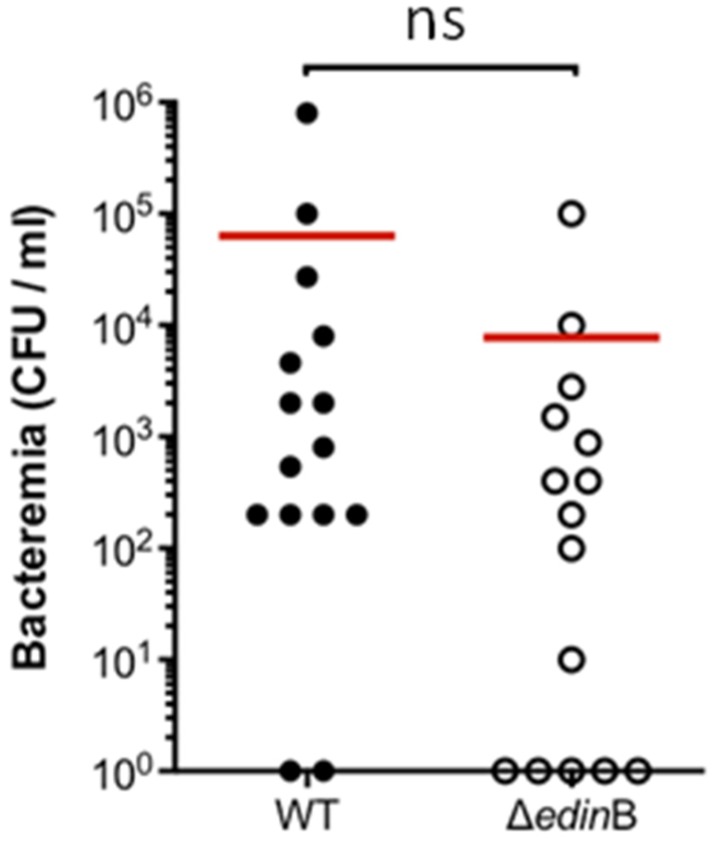
Measure of bacterial persistence in the bloodstream. Groups of mice (*n* = 15 each) were infected by intravenous inoculation of 3 × 10^7^ CFU of the WT and Δ*edin*B strains. Dots represent the CFU values in the bloodstream for each animal at 48 h post infection; red bars show the mean values; ns, not significant.

Finally, we aimed at determining whether the presence of *edin*B might favor bacterial persistence in the bloodstream. Two groups of mice (*n* = 15) were injected intravenously with either WT or the Δ*edin*B strain. We recorded the bacterial burden in the blood at 48 h of infection. This analysis revealed no significant difference between the bacterial burdens in both groups of infected animals (Figure 5).

Expression of EDIN-B does not affect bacterial persistence in the blood per se.

## 3. Discussion

Hospital- and community-acquired methicillin-resistant *S. aureus* is an increasingly recognized etiology of pneumonia and presents diagnostic and therapeutic challenges. In particular, the pejorative evolution of this type of infection into deadly bacteremia has clinical relevance [1,2]. Here, we report that ST80-MRSA-IV expressing EDIN-B produced a high occurrence of bacteremia in animals suffering from pneumonia. In a mouse model of infection, we demonstrate the critical role of EDIN-B activity in the translocation of bacteria from the lung tissues to the bloodstream, without excluding a contributing role of additional virulence factors of *S. aureus*. We also found that *edin*B does not promote detectable bacterial persistence in the lung tissues or in the blood. These results deserve further attention to confirm a similar role in humans given that human cells are sensitive to EDIN cytotoxicity. Collectively, our data point f a major role of EDIN*-*B as a virulence factor of methicillin-resistant *S. aureus* ST80 and for a possible implication of EDIN in pneumonia-induced bacteremia.

EDIN belongs to a large family of exoenzymes that target the small GTPase RhoA for inactivation [7,10]. The small GTPase RhoA is an essential regulator of actomyosin cytoskeleton contractility, thereby controlling cell adhesion, division, and migration [20]. We did not record a difference in bacterial persistence in the lung tissues or in the bloodstream, most likely excluding a role for EDIN at the level of immune cell effectors. Our data rather favor the hypothesis of a role for EDIN at the level of the progressive translocation of the bacteria from the lung tissues to the bloodstream. Indeed, by promoting the assembly of contractile actin cables, the small GTPase RhoA exerts a critical role in the control of barrier function of epithelia and endothelia that is a hotspot of targeting by bacterial virulence factors [5,15,18,20]. In contrast, when we injected bacteria in the bloodstream, we did not detect a specific role for EDIN-B in promoting their extravasation to the lung tissues (data not shown). Instead, we provide compelling evidence that EDIN-B activity promotes the translocation of bacteria from the lung mucosa to the blood. This breaching of host barriers may occur via different scenarios, such as a higher capacity of the bacteria to transcytose or to reduce the cohesion of the lung epithelial and endothelial barriers [19]. This second scenario is in agreement with cell biology approaches that have firmly established that RhoA inactivation contributes to notch intercellular junctions in epithelia [15,18]. More recent studies also documented the capacity of EDIN factors to induce the formation of transcellular tunnels in endothelial cells [19]. Consistent with these findings, we also observed a requirement of EDIN-B in the induction of macroapertures during endothelial cell infection (Figure S1). Further studies will have to clarify whether EDIN-B promotes the translocation of bacteria through two host barriers in the lung successively by intercellular and transcellular pathways.

Infection by pathogenic strains of *S. aureus* involves the deployment of a large arsenal of virulence factors [3,4]. Despite this diversity, we identified a critical role for EDIN in promoting bacterial dissemination. This work represents the first demonstration that EDIN-B is a virulence factor and thus fulfills the molecular Koch’s postulates. This study also reveals a major role of EDIN in promoting the translocation of bacteria from the mucosal environment to the blood circulation. These data extend previous findings suggesting that RhoA ADP-ribosyltransferase activity promotes the metastatic dissemination of *S. aureus* via a hematogenous route in mice [15,21] and that in humans, *edin-*positive strains of *S. aureus* are found in deep seated infections, such as high-grade diabetic foot ulcerations associated with bacteremia [6,22].

Our study in mice provides a genetic demonstration of the invasive virulence function of EDIN activity.

## 4. Experimental Section

### 4.1. Ethics Statement

The mice were maintained and handled in strict accordance with the recommendations for the ethical evaluation of experiments using laboratory animals and with the European guidelines 86/609/CEE. This protocol was approved by the ethics committee for animal experimentation (CIEPAL AZUR/N°28) and by the French Ministère de l’Education Nationale, de l’Enseignement Supérieur et de la Recherche under number 02330.01.

### 4.2. Bacterial Strains and DNA Constructs

HT20020209-LUG1799 is a minimally passaged ST80 *SCCmecIV* PVL + MRSA that was originally isolated in France [23] and that is referred to as WT in this study. All strains *of S. aureus* belonging to this lineage, such as HT20020209-LUG1799, contain *edinB* only [24]. Deposit in process to EMBL and http://www.ebi.ac.uk/ena. The isogenic Δ*edin*B mutant (LUG1840) was obtained by allelic replacement mutagenesis in LUG1799 using the pMAD plasmid as previously described [25], except the tetracycline resistance gene was replaced by a kanamycin resistance gene from the plasmid PCR 2.1 (Invitrogen, Carlsbad, CA, USA). The primers used to amplify the upstream and downstream regions of *edin*B were as follows: edin1117′, edin2165, edin2872, and edin3795 (Table S1). The primers used to verify the deletion of *edin*B were edinB-1 and edinB-2 (Table S1, Figure 1A). Replacement in the LUG1799 strain of the edin gene by the kanamycin cassette was confirmed by PCR using primers edin1099-Kcr4 and Kcr3-edin3893 (Table S1, data not shown). LUG1840 mutants were also complemented with either *edin*B-WT (Δ*edin*B/*edin*B-WT) or *edin*B-RE, which encodes for the catalytically inactive form of EDIN-B (Δ*edin*B/*edin*B-RE). The coding region of *edin*B was amplified by PCR using genomic DNA from LUG1799 and the primers *edin*B*-*F and *edin*B*-*R (Table S1). The *Bam*HI-*Pst*I PCR product was cloned into the vector pMK4-pPROT as described previously [26]. The resulting plasmid pMK4-pPROT-*edin*B was maintained in *Escherichia coli* Top10 (Invitrogen, Carlsbad, CA, USA), subcloned into *S. aureus* RN4220 and then transferred into LUG1840 mutants by electroporation. The EDIN-B R185E mutation was introduced by changing AGA to GAA in the coding sequence of *edin*B in pMK4-pPROT-*edin*B using a QuikChange Lightning Site-Directed Mutagenesis Kit (Agilent Technologies, Les Ulis, France). The recombinant plasmids were verified by sequencing. The bacterial strains were grown in Luria-Bertani medium alone or supplemented with kanamycin (50 µg/mL) or chloramphenicol (20 µg/mL) at 37 °C. We verified that all strains had identical growth properties (data not shown).

### 4.3. Biochemical Assays and Products

For immunoblotting, serum anti-EDIN was raised using standard rabbit immunization protocols (Agro-Bio, La Ferté Saint Aubin, France). Primary antibodies were revealed using goat anti-rabbit horseradish peroxidase-conjugated secondary antibodies (Dako, Glostrup, Denmark), followed by chemiluminescence detection ECL (GE Healthcare, Little Chalfont, UK). For RhoA ADP-ribosylation assays, each reaction was performed in a total volume of 50 μL, 30 min at 37 °C with 0.8 μg of RhoA, 10 μM biotinylated NAD^+^ (R & D Systems) and 1 μL of supernatant in 50 mM Tris-HCl (pH 7.5), 0.1 mM DTT, 1 mM MgCl_2_, 100 mM NaCl and complete protease inhibitors (Roche, Manheim, Germany). The reaction was stopped by adding 5× Laemmli buffer (Sigma, St Louis, MO, USA) and by boiling the samples for 5 min. The samples were further subjected to SDS-PAGE and transferred to PVDF membranes. The biotin-ADP-ribosylated proteins were visualized with peroxidase-coupled streptavidin (Sigma, St Louis, MO, USA) in a subsequent chemiluminescence reaction.

### 4.4. Murine Model of Pneumonia and Bacteremia

Six to eight-week-old female BALB/c mice were purchased from Janvier Laboratory (Le Genest St. Isle, France). Before infection, the bacterial strains were grown in LB medium with shaking for 16 h. The cultures were centrifuged, washed three times in phosphate-buffered saline (PBS; Gibco, Grand Island, NE, USA) and diluted in PBS to achieve the desired inoculum. Pneumonia was triggered by a 20-μL injection in each nostril for a total inoculum of approximately 10^10^ bacteria/mouse in animals anesthetized by intraperitoneal injection of xylazine (10 mg/kg) and ketamine (90 mg/kg). The bacterial burden in the blood was assessed after 5 µL of blood was collected, diluted in PBS and spread on LB agar plates with the corresponding antibiotic. The colony forming units (CFUs) were counted at 24 h after incubation at 37 °C. At the indicated period, the mice were euthanized, and the lungs were collected, weighed and homogenized in PBS using a Precellys 24 homogenizer before CFU enumeration. For bacteremia, the mice were directly infected with approximately 10^7^ bacteria/mouse by intravenous injection of 100 µL of inoculum in the tail vein. The level of bacterial burden was recorded as indicated above.

### 4.5. Histopathology

The lungs from control and infected mice were collected, fixed in formalin and embedded in paraffin. Consecutive 3-µm paraffin sections were stained with hematoxylin-eosin-saffron (HES). The presence of bacteria within lung tissues was visualized using rabbit anti-*S. aureus* primary antibody (Eurogentec, Angers, France) and goat secondary anti-rabbit HRP antibody (Dako, Glostrup, Denmark).

### 4.6. Cell Culture and Immunofluorescence

Human umbilical vein endothelial cells (HUVECs, Promocell) were cultured in SFM medium (Invitrogen), supplemented with 20% Fetal Calf Serum (Invitrogen), 20 ng/mL fibroblast growth factor, 10 ng/mL epidermal growth factor, 0.01 U/mL penicillin, 10 ng/mL streptomycin and 1 µg/mL heparin (Sigma) For bacterial infection assays, 25,000 cells per well were seeded on gelatin coated (0.4% *w*/*v* in H_2_O) glass coverslips in 12-well plates 24 h before infection. After infection, cells were fixed in 4% formaldehyde (Sigma) in PBS. Actin filaments were labeled using 1 µg/mL FITC-conjugated phalloidin (Sigma). Bacteria were labeled with rabbit serum anti-*S. aureus* (Eurogentec). Texas Red anti-rabbit IgG antibody (Molecular Probes, ThermoFisher Scientific, Waltham, MA, USA). Immunosignals were analyzed with a DM5500 microscope (Leica, Wetzlar, Germany) with a ×63 lens.

### 4.7. Statistical Analysis

Statistical analyses were performed using PRISM V 5.0b (GraphPad Software, San Diego, CA, USA). For the pairwise analysis, a Mann-Whitney non-parametric test was performed; *p-*values <0.05 were considered statistically significant.

## Supplementary Materials

Supplementary materials can be accessed at: http://www.mdpi.com/2072-6651/7/10/4131/s1.
