# Supplementary material for: EDIN-B Promotes the Translocation of Staphylococcus aureus to the Bloodstream in the Course of Pneumonia
Source: Toxins (Basel). 2015 Oct 15;7(10):4131–42. doi: 10.3390/toxins7104131 (PMC4626725; doi:10.3390/toxins7104131)
Supplement: Supplementary file 1 [file toxins-07-04131-s001.pdf]

Supplementary Data

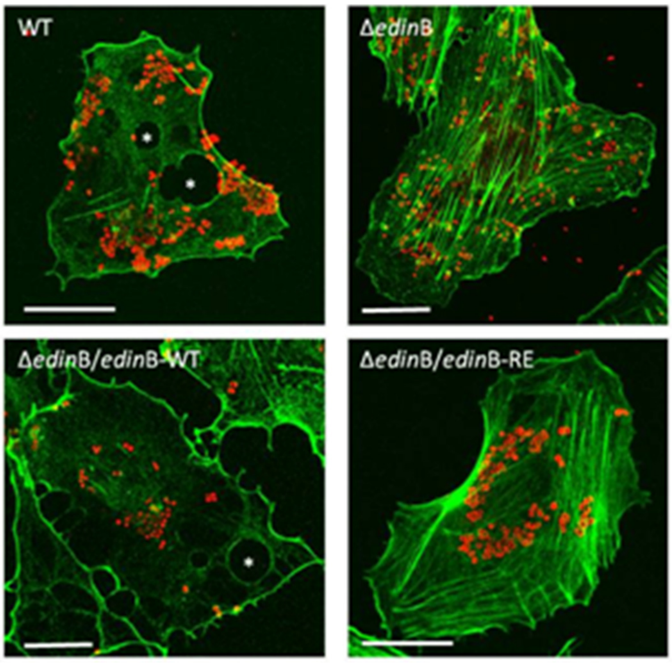

Figure S1. ST80 expressing EDIN induces transcellular macroapertures. HUVECs were infected 2 h at a MOI of 100 with the wild-type strain ST80 *SCCmecIV* PVL + MRSA (ST80), the isogenic strain deleted from *edin*B (Δ*edin*B) and the Δ*edin*B strain complemented with expression plasmids encoding EDIN WT (Δ*edin*B/*edin*B-WT) or the catalytically inactive mutant R185E (Δ*edin*B/*edin*B-RE). Visualization of the actin cytoskeleton with FITC-conjugated phalloidin (green) and bacteria using a rabbit serum anti-*S. aureus* and Texas Red-conjugated anti-rabbit IgG (red). Stars indicate macroaperture location. Bars = 10 µm.

Table S1. Primers used in this study.

Primer Names	Primer Sequences
edin1117	5′-GGAGGATCCGGAGGAAAATATAATTTACCGATAGG-3′
edin2165	5′-CTCCTCGAGCATATTTAAATTCATCTCCTATGATA-3′
edin2872	5′-CTCCTCGAGTAACTGCTGTAGTTTTTAAAAAATAAG-3′
edin3795	5′-GGAGGATCCAACCTATATGAGAAATTTTTGGATATTTC-3′
edinB-F	5′-GGGGATCCATGAAAGATACAATTGTAAAATTTCTATCAGCATTCCTTGTG-3′
edinB-R	5′-GGCTGCAGTTATTTTTTAAAAACTACAGCAGTTATAATTATCTTTCTTTTG-3′
edinB-1	5′-GACTAGTTGAAGCTACTAAATGGGG-3′
edinB-2	5′-CAGCATATTCTGTCCCTCTAGG-3′
edin1099	5′-GAGTTAGTTGGTATTCATGTTGG-3′
Kcr4	5′-CTATCGCCTTCTTGACG-3′
Kcr3	5′-CGTTTCTGCGGACTGG-3′
edin3893	5′-CAGTAAAATGCATTTATTCGGTGG-3′

Restriction sites are underlined.
